# New Application of Iodin

**Published:** 1899-12

**Authors:** 


### New Application of Iodin.

Elsberg recommends a 20 percent solution of iodin in equal parts of alcohol and ether as the best local application of this agent. He has in no case seen any iodin-poisoning from its use, and in one only did he obtain a well-marked iodin reaction in the urine. It has the advantage of rapidly drying, and only one coat need be painted on the part. He believes that therapeutic results can be obtained with it which can not be obtained with the much weaker official tincture of iodin commonly employed.—*The Journal.*

